# Sequencing the CHO DXB11 genome reveals regional variations in genomic stability and haploidy

**DOI:** 10.1186/s12864-015-1391-x

**Published:** 2015-03-08

**Authors:** Christian Schrøder Kaas, Claus Kristensen, Michael J Betenbaugh, Mikael Rørdam Andersen

**Affiliations:** Mammalian Cell Technology, Global Research Unit, Novo Nordisk A/S, A9.2.36, Novo Nordisk Park, 2760, Måløv, Denmark; Network Engineering of Eukaryotic Cell Factories, Technical University of Denmark, Kgs Lyngby, Denmark; Chemical and Biomolecular Engineering, Johns Hopkins University, Baltimore, MD USA; Institute of Cellular and Molecular Medicine, University of Copenhagen, Copenhagen, Denmark

**Keywords:** Copy number variations (CNVs), CHO DXB11, CHO cells, *C. griseus*, Single nucleotide polymorphisms (SNPs)

## Abstract

**Background:**

The DHFR negative CHO DXB11 cell line (also known as DUX-B11 and DUKX) was historically the first CHO cell line to be used for large scale production of heterologous proteins and is still used for production of a number of complex proteins.

**Results:**

Here we present the genomic sequence of the CHO DXB11 genome sequenced to a depth of 33x. Overall a significant genomic drift was seen favoring GC → AT point mutations in line with the chemical mutagenesis strategy used for generation of the cell line. The sequencing depth for each gene in the genome revealed distinct peaks at sequencing depths of 0x, 16x, 33x and 49x coverage corresponding to a copy number in the genome of 0, 1, 2 and 3 copies. This indicate that 17% of the genes are haploid revealing a large number of genes which can be knocked out with relative ease. This tendency of haploidy was furthermore shown to be present in eight additional analyzed CHO genomes (15-20% haploidy) but not in the genome of the Chinese hamster. The *dhfr* gene is confirmed to be haploid in CHO DXB11; transcriptionally active and the remaining allele contains a G410C point mutation causing a Thr137Arg missense mutation. We find ~2.5 million single nucleotide polymorphisms (SNP’s), 44 gene deletions in the CHO DXB11 genome and 9357 SNP's, which interfere with the coding regions of 3458 genes. Copy number variations for nine CHO genomes were mapped to the chromosomes of the Chinese hamster showing unique signatures for each chromosome. The data indicate that chromosome one and four appear to be more stable over the course of the CHO evolution compared to the other chromosomes thus might presenting the most attractive landing platforms for knock-ins of heterologous genes.

**Conclusions:**

Our studies reveal an unexpected degree of haploidy in CHO DXB11 and CHO cells in general and highlight the chromosomal changes that have occurred among the CHO cell lines sequenced to date.

**Electronic supplementary material:**

The online version of this article (doi:10.1186/s12864-015-1391-x) contains supplementary material, which is available to authorized users.

## Background

The global market for biopharmaceuticals is currently 140 billion USD of which the majority of proteins requiring post-translational modifications are produced in Chinese Hamster Ovary (CHO) cells [1]. CHO cells have a long history as a production organism in industry due to their ability to grow in suspension without serum and to be scalable to large production volumes. Furthermore, CHO cells are able to produce proteins with a glycosylation pattern similar to that of humans [2] and are not infected by a wide range of viruses dangerous to humans [3]. So far more than 40 biopharmaceuticals including monoclonal antibodies, hormones, cytokines and blood-coagulation factors have been produced in CHO cells.

The CHO cell line was originally isolated in 1957 by T. Puck [4] and ten years later the CHO-K1 cell line was derived from this ancestral host [5]. In order to facilitate creation of stable cell lines producing a gene of interest a selection system was needed. The CHO DXB11 cell line was created with the goal of developing a stable CHO cell line with a DHFR negative phenotype as DHFR can catalyze the conversion of dihydrofolic acid to tetrahydrofolic acid – an essential cofactor carrier. CHO-K1 cells were first exposed to a round of random chemical mutagenesis using Ethyl methanesulfonate (EMS) to generate the UKB25 cell line (*dhfr*+/*dhfr*-) [6] followed by a second round of mutagenesis using γ-radiation before isolation of the CHO DXB11 cell line (*dhfr*-/*dhfr*-) [7]. The DXB11 cell line was not mentioned by name in the original paper [6] and the name was not published until 1982 where the gene structure was more thoroughly investigated [7]. During the period between these two papers other laboratories used the cell line under the names CHO K1 DUX-B11 [8] and DUKX-CHO [9], explaining the origin of other names commonly used to describe the CHO DXB11 cell line. Further details concerning the clonal history of the CHO cell lines can be found in a recent review [10]. Historically, CHO DXB11 was the first CHO host cell for large scale production of a protein product (human tissue plasminogen activator [10,11]) and it is still being used for production of several protein products on the market.

Recently the genomic sequence of the Chinese hamster (*C. griseus*) [12,13] and seven CHO cell lines [12] were released making genomic comparisons of CHO cells possible for the first time. The first attempt to analyze the genomic information of the CHO DXB11 cell line was done in 2005 when Wlaschin *et al.* extracted 4608 expressed sequencing tags from CHO DXB11 RNA in order to create a CHO specific cDNA microarray [14]. This work furthermore lead to sequencing of the CHO mitochondrial genome. A 1x coverage of the genome of a CHO DXB11 transfectant producing human secreted alkaline Phosphatase was released back in 2011 [15] the same year as the CHO-K1 ATCC sequence was made public [3]. They furthermore reported that the *dhfr*-gene was detected albeit showing low coverage.

In this work, the genome of the CHO DXB11 cell line was sequenced with the goal of making this genome publicly available alongside the list of previously sequenced CHO genomes [12]. The genome was analyzed in order to validate the genomic cause of the DHFR negative phenotype of the cell line and the overall genome composition was compared to the currently sequenced CHO genomes. We found unique patterns for the evolution of each of the chromosomes from the Chinese hamster to each of the CHO cell lines and a surprising degree of haploidy.

## Results

### Sequencing depth per gene predicts gene haploidy and polyploidy

**Figure 1:**
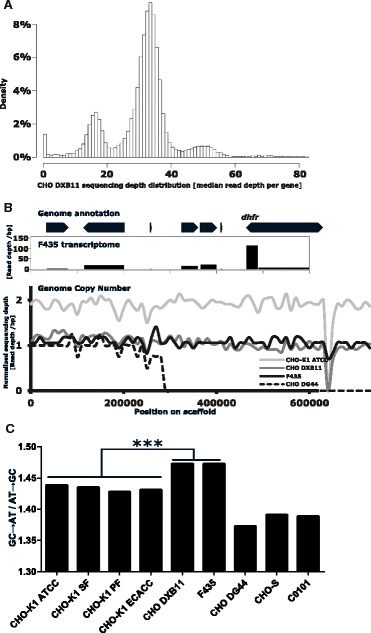
**Copy number distribution for the CHO DXB11 genome. A)** Distribution of the sequencing depth of the 20661 genes in the CHO DXB11 genome. District peaks are seen at depth of 0x, 16x, 33x and 49x corresponding to a copy number in the genome of 0, 1, 2 and 3 copies. **B)** Sequencing depth of the scaffold carrying *dhfr*. The entire 0.7 Mb scaffold is seen to be present in two copies in CHO-K1 ATCC, one copy in CHO DXB11 and roughly half the scaffold was deleted completely in CHO DG44. A peak in mRNA levels indicates that *dhfr* is seen to be transcriptionally active in F435 from the one allele that still contains the gene. **C)** The ratio of GC → AT and AT → GC mutations in CHO cell lines versus the *C. griseus* genome. A significant bias towards GC point mutations are observed in the two CHO DXB11 cell lines compared to the four CHO-K1 cell lines. Statistical significance level: ***p < 0.001.

In order to gain insight into the genotype of the industrially relevant cell line CHO DXB11, as well as addressing some of the genomic consequences associated with creating a stable CHO transfectant the two genomes were sequenced. Genomic DNA was extracted from adherently growing CHO DXB11 cells as well as F435 cells, which is CHO DXB11 cells transfected with the gene encoding Coagulation Factor VIII [16] and subsequently adapted to suspension culture growth. Both cell lines were sequenced using the Illumina HiSeq 2000 platform yielding 0.5 bn paired reads for CHO DXB11 and 0.3 bn reads for F435. The reads were aligned to the Chinese hamster genome with a median depth of 33x and 17x respectively. The sequencing depth in CHO DXB11 for each of the 20661 genes found in the *C. griseus* genome were calculated and plotted, showing distinct peaks at sequencing depths of 0x, 16x, 33x and 49x coverage corresponding to a copy number in the genome of 0, 1, 2 and 3 copies (Figure 1A). The *dhfr* gene was found at a depth of 2.19 in the CHO-K1 ATCC genome, 0 in the CHO DG44 genome, 1.29 in the F435 genome and 1.06 in the CHO DXB11 genome in accordance with one allele being lost by gamma radiation in CHO DXB11 (L.A. Chasin, personal communication). Seven other genes found flanking *dhfr* on the same scaffold are also seen to be present in only one copy in the genomes of CHO DXB11 and F435. The *dhfr* gene from the remaining allele is found to be transcribed as seen from RNA sequencing data from F435 (Figure 1B) but contain a homozygous G410C point mutation located in the *dhfr* coding region causing a Thr137Arg missense mutation. This threonine is conserved from *C. elegans* to mouse, rat, hamster and human. In the crystal structure of murine DHFR [17] (96.3% similarity) the threonine is found in the cleft binding dihydrofolic acid right next to the active site, thus supporting the hypothesis that this mutation is able to effectively inactivate DHFR. In the process of deleting *dhfr* in the CHO DG44 genome it is found that four of the flanking genes were deleted (Zfyve16, Fam151b, Ankrd34b and Msh3) (Figure 1B).

### A significant drift in single nucleotide polymorphisms is observed

In addition to the single nucleotide polymorphism (SNP) found in the *dhfr* gene, a total of 2,496,390 SNPs were found in the CHO DXB11 genome when aligned to the *C. griseus* genome (Table 1). For the CHO-K1 ATCC genome a higher total number of SNPs were detected but more SNPs were found in the coding regions of the CHO DXB11 genome (Table 1). 91% of the mutations interfering with translation in CHO-K1 ATCC were also found in CHO DXB11. All SNPs found in CHO-K1 ATCC and CHO DXB11 genes are listed in Additional file 1: Tables S3 and S4.

**Table 1:** **Overview of SNPs and indels in the CHO DXB11 and CHO-K1 ATCC genome**

	**CHO K1 ATCC**	**CHO DXB11**
SNPs	2,527,490	2,496,390
Intronic SNPs	639,171	636,613
SNPs in CDS regions	19,096	21,142
SNPs missense/nonsense	8,195	9,357
Indels	341,848	315,422
Indels in CDS regions	211	259
Frameshifting indels	170	197

By comparing all the SNPs from the available CHO genomes to that of *C. griseus* a significant drift favoring GC → AT point mutations is evident for the two CHO DXB11 cell lines compared to the four sequenced CHO-K1 cell lines (Figure 1C), probably caused by the chemical mutagen used in the creation of the CHO DXB11 cell line. However, different SNP biases were seen for CHO DG44 and CHO-S/C0101 respectively probably due to the distinct evolution of these cell lines (Figure 1C).

### Copy number variation signature is chromosome dependent

**Figure 2:**
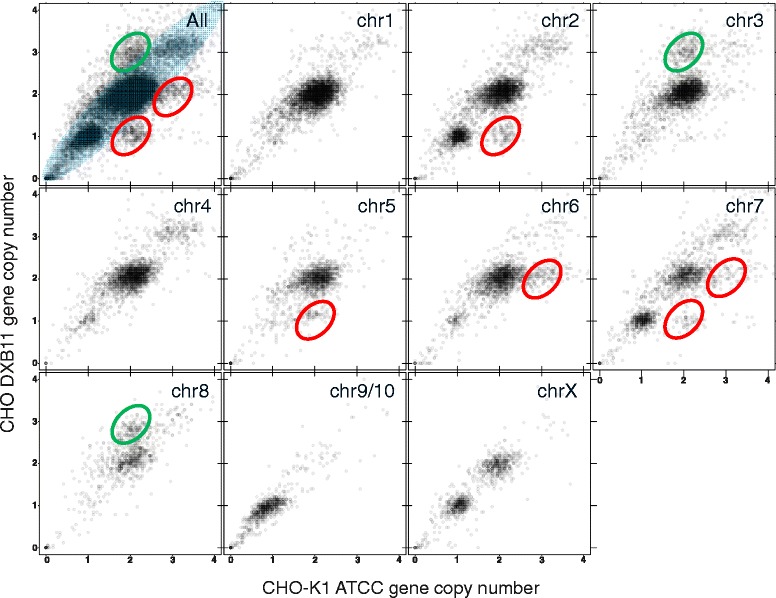
**Copy number plot for the CHO-K1 ATCC versus CHO DXB11 genomes.** The normalized sequencing depth of each gene in the *C. griseus* genome as found in CHO-K1 ATCC and CHO DXB11 listed by chromosome. Top left plot shows the distribution across all chromosomes. Red circle indicate genes, which have been reduced in copy number (e.g. from diploid to haploid), a green circle indicate duplication of genes and the blue circle in the top left plot indicate genes without change in copy number.

By comparing the sequencing read depth for each gene from the CHO-K1 ATCC to the CHO DXB11 genome, it is seen that 96% of the genes are found in similar depths in both cell lines as expected, following the diagonal of the plot in Figure 2. No genes are observed to be deleted in the CHO-K1 ATCC genome but present in the CHO DXB11 genome as expected due to the origin of the CHO DXB11 cell line from CHO-K1. However, a total of 506 genes are seen to have reduced copy numbers in the CHO DXB11 genome compared to the CHO-K1 ATCC genome, whereas 389 genes have increased copy number. The copy number data for the CHO-K1 ATCC and the CHO DXB11 genome were furthermore mapped to the individual chromosomes of *C. griseus* (Figure 2) revealing a unique signature for each chromosome differing from the signature of the genome as a whole. It was not possible to separate chromosome nine from chromosome ten when sequencing the *C. griseus* genome and for this reason these two are listed together [13]. On chromosome two, where *dhfr* is situated, 89 genes have been reduced from diploid to haploid. Reductions are furthermore seen on chromosome five, six and seven. On chromosome three 158 genes are found to be triploid in CHO DXB11 versus diploid in CHO-K1 ATCC.

**Figure 3:**
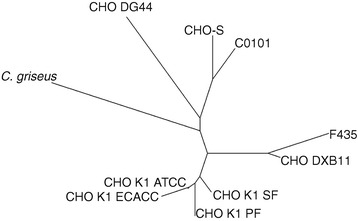
**Phylogenetic tree based on copy number variation between cell lines.**

**Figure 4:**
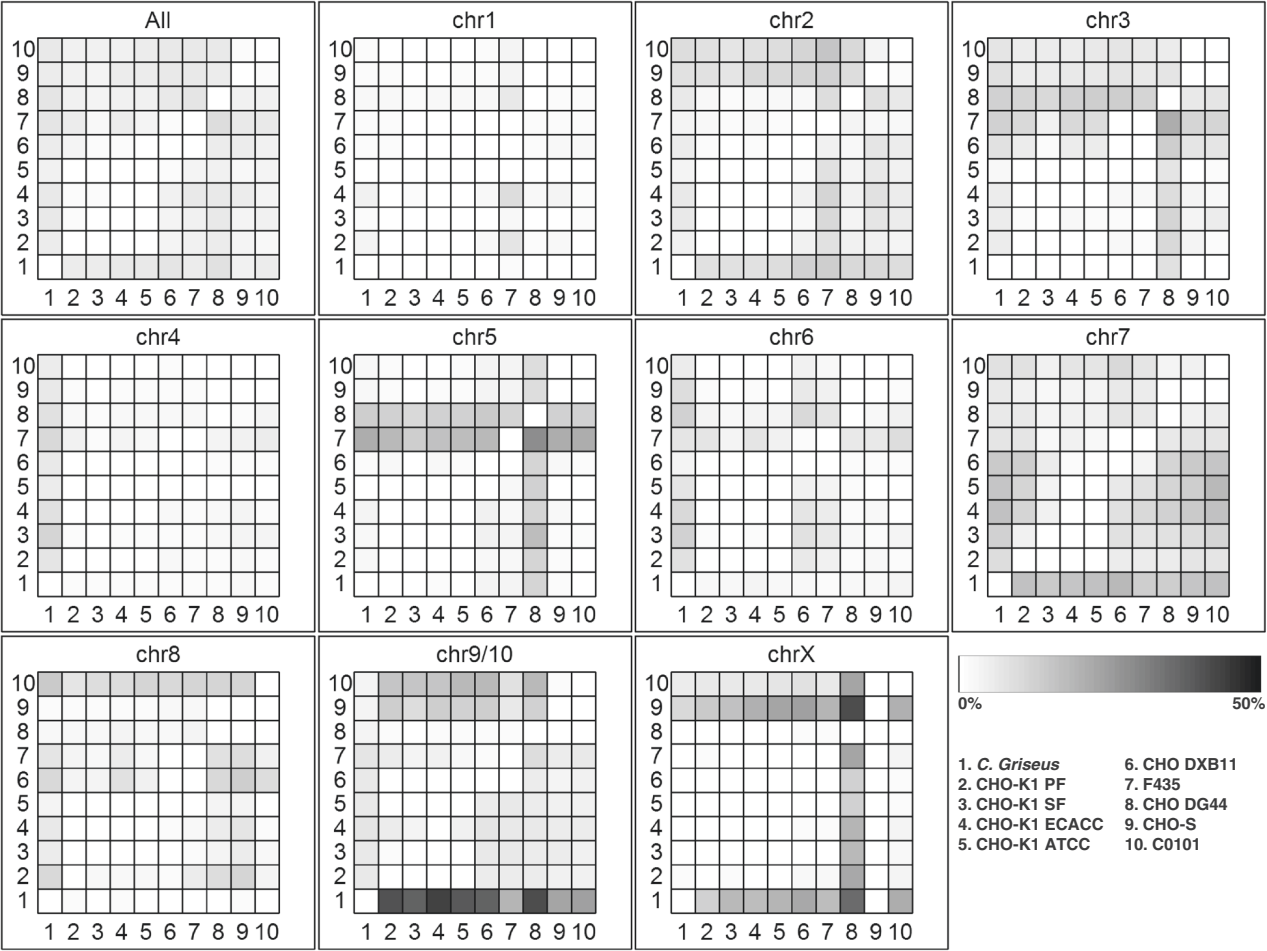
**Heat maps showing the copy number variations among the 10 analyzed genomes.** The intensity of coloring indicate the percentage of genes found on the given chromosome that has undergone a copy number reduction. Reduction in copy number in all cell lines except one is revealed by a vertical line and amplification of genes as a horizontal line.

By estimating the extent of copy number variations (CNVs) between the currently sequenced CHO cell lines, a phylogenetic tree can be drawn, which accurately recapitulates the overall cell line history [10] (Figure 3). A heat map showing the extent of genes found to have reduced copy numbers between the different cell lines reveal specific patterns for each chromosome (Figure 4). From this, it can be seen that each chromosome is shown to have evolved differently across the cell lines and exhibits unique patterns. On chromosome 9/10, ~70% of the genes have been reduced from diploid in *C. griseus* to haploid in all cell lines except for C0101, CHO-S and F435 where only ~50% of the genes are haploid (Additional file 2: Figure S3). Chromosome X is seen to contain only 9% haploid genes in CHO-S whereas ~70% of the genes on this chromosome are found to be haploid in CHO DG44. Chromosome five appears to have undergone changes especially in F435 (amplification) and in DG44 (amplification of some genes and reduction of others) (Additional file 2: Figure S2). Chromosomes one and four seem to be the most stable chromosomes for all the CHO cell lines. Overview of the number of genes found to be deleted, haploid, diploid or amplified in each genome are listed in Table 2.

**Table 2:** **Overview of copy number estimation in the sequenced cell lines**

	**CN = 0**	**CN = 1**	**CN = 2**	**CN > 2**
*C. griseus*	0	0	20,661	0
CHO-K1 ATCC	30	3,773	15,305	1,553
CHO-K1 ECACC	57	4,039	13,310	3,255
CHO-K1 PF	54	3,356	15,059	2,192
CHO-K1 SF	48	3,073	15,453	2,087
CHO DXB11	44	3,586	15,267	1,764
F435	53	3,888	14,306	2,414
CHO DG44	62	4,219	13,967	2,413
CHO-S	47	3,024	15,603	1,987
C0101	37	3,544	15,088	1,992

In addition to chromosome specific changes in copy number it was investigated whether specific gene functions were seen to be enriched among genes with CNV’s. 135 Gene Ontology (GO) terms were found to be significantly enriched with either amplified for reduced genes compared to *C. griseus*. The GO-terms were found to be conserved among all nine CHO cell lines (Additional file 1: Table S7, Additional file 2: Figure S4). The 3rd most significant GO-function were genes with transcription factor activity where CHO DXB11 and F435 had amplification of 49 and 51 genes respectively out of 432, whereas the other 8 CHO cell lines have 27 ± 3 genes amplified in this category. In the same category CHO DG44 were found to have 36 genes which had been reduced in copy number where the remaining eight cell lines had 16 ± 2 genes reduced.

## Discussion

The genetic drift of SNPs from *C. griseus* to CHO DXB11 exhibited a significant disparity compared to the drift from *C. griseus* to the four CHO-K1 cell lines. The drift can likely be explained by the fact that the CHO DXB11 cell line was treated with the chemical mutagen EMS. The ethyl group of EMS is able to alkylate guanine forming O-6-ethylguanine which during replication commonly is paired with thymine and not cytosine [18,19]. Thus, an increased number of GC → AT mutations are expected and found in the genome of this cell line. It was seen that *dhfr* contained a missense mutation as well as the loss of an allele. These findings confirm observations by Lawrence Chasin in 1982 (personal communication, unpublished results). The threonine on position 137, which is mutated into an arginine in DXB11, is not found in any of the annotated domains of the protein but is found to be highly conserved. The amino acid is located close to the active site and it can thus be hypothesized that the polar arginine is able to interfere with the structure of the binding cleft leading to inactivation of the enzyme. Due to the fact that only one allele of *dhfr* has been deleted and the other is transcriptionally active, it should be possible to find revertants as background in a transfection experiment. Indeed, revertants have been detected albeit at a frequency less than 10e-8 (L.A. Chasin, personal communication).

SNPs are the most frequent type of genetic polymorphism found when resequencing genomes from a common ancestor [20] and single mutations in the coding regions can result in significant changes for the phenotype of the cell lines. For sequenced diploid genomes without known and validated SNPs, it has previously been seen to filter out SNPs with a depth less than half the mean depth of the genome [12]. But in the case of CHO DXB11 (or any of the other sequenced CHO cell lines) that practice would result in SNPs found in most haploid genes (17% of the genes) to be remain undetected. For that reason a more lenient filter has to be applied in the current study for homozygous SNPs, which can be found in the haploid genes, and a more stringent filter requiring higher depth for heterozygous SNPs. The need to check for mutations in the relevant cell line before designing a laborious knock down, knockout or PCR primer solely based on the *C. griseus* genome sequence is highlighted by the 9357 SNPs, which were found in the CHO DXB11 genome within the coding regions of 3458 genes. All genes containing SNPs as well as genes containing indels for CHO-K1 ATCC and CHO DXB11 are listed in Additional file 1: Table S2. SNPs located in the other sequenced cell lines are not included due to the fact that the current lower sequencing depth of these resulted in a ~20% probability of a correct SNP call versus >99% in CHO DXB11 and K1 ATCC.

The copy number of a gene has previously been found to correlate well with the sequencing depth [21,22] and this correlation was used to determine the copy number of the 20661 genes both in the *C. griseus* genome and in the nine sequenced CHO cell lines. Based on analysis of the sequencing depth per gene it was found that only wild type *C. griseus* had a single peak centered on a copy number of two whereas the sequenced CHO cell lines derived from this organism show distinct peaks revealing large number of genes only present in one copy or amplified to three or more copies. Interestingly, the data indicates that 15-20% of the genes found in the nine CHO cell lines sequenced to date are haploid. Historically, the CHO cells were often regarded functionally haploid at many genetic loci making these cell lines ideal for investigation of molecular functions in a eukaryotic cell model [14,23,24]. This current data explain this perceived haploid phenotype and this knowledge can also be advantageous for choosing a knock-out target in a specific pathway or for elucidating target region for a knock in. As chromosome one and four have the lowest level of CNVs across the nine CHO cell lines, some of which have encountered heavy mutation pressure over a long period of time [10], these chromosomes may be considered attractive landing platforms for knock-ins of heterologous genes.

Based on the current sequencing data from CHO DXB11 and available data from other CHO cell lines a phylogenetic tree was created based on the copy variations between the cell lines. The tree reflects the clonal history of the cell lines (see recent review for details [10]) and correlates well with a phylogenetic tree recently published based on SNPs [12]. The CHO-K1 cell lines lie in one cluster neighboring the CHO DXB11 cluster and distant from the CHO DG44 and CHO-S/C0101 branches. The clonal history of the F435 cell line by transfection of CHO DXB11 is apparent from the phylogenetic tree as F435 emerges out of the same branch as CHO DXB11 (Figure 3). Nonetheless, a total of 907 genes are found to have undergone a CNV in the process from transfection of a pool of CHO DXB11 cells, amplification of the insert and subsequent adaptation to suspension culture growth.

To give a more precise estimate of the genomic differences the sequencing depth across the coding regions of the genes (1.7 kb on average) were measured. This allowed for normalization using hundreds of sequencing reads compared to looking at SNPs only supported by a dozen or so reads at most. Furthermore, the coding DNA sequences (CDS’s) are the most uniquely defined elements of the genome. For this reason, assessment of the CNVs are able to provide a more detailed look into the cell lines even when only very low sequencing depths are available. This is highlighted by the clustering of the CHO-K1 cell lines closely together, even though CHO-K1 ATCC has been sequenced to a depth of 45x which is ~6x that of the other three CHO-K1 cell lines and C0101 is sequenced to a depth 3x that of CHO-S.

Each chromosome showed a distinct signature of CNVs giving for the first time an insight into CHO chromosomal genome stability from next-generation sequencing data. In the future this method could be used in combination with FISH to validate hypotheses on e.g. the range of genetic reductions and rearrangements on a particular chromosome (e.g. chromosome two containing *dhfr*) from CHO-K1 ATCC to CHO DXB11. The method also revealed a large number of haploid genes on chromosome 9/10 which seem to have been reduced in the earliest CHO cell lines prior to evolving into the cell lines sequenced today. Some of these mutations might have been critical for establishing the independent immortal first CHO cell lines.

Each rearrangement event that has occurred in the evolution from CHO-K1 to CHO DXB11 may have had an impact on a multitude of genes as seen by all eight genes on the scaffold holding *dhfr* were found to be haploid (Figure 1B) but probably caused by one single deletion event during UV radiation. Therefore, the number of genes with altered CNVs is no true indication of the number of genomic rearrangements that has occurred as one large rearrangement events could impact dozens of genes. Due to the short lengths of the genomic scaffolds in the current versions of the *C. griseus* and CHO-K1 genomes, it is not yet feasible at this time to piece together the rearrangement history of the CHO cell lines, but 3rd generation sequencing could permit the construction of a *C. griseus* genome with a reduced number of scaffolds [25]. Once a more complete scaffold is available, CNV data could be used to make genomic based chromosomal maps which currently are only done using FISH [26-28].

With improved genome constructions possible in coming years, it will be informative to elucidate more detailed genomic differences between CHO DG44 and CHO DXB11 as they are the two DHFR negative CHO cell lines most widely used today. As described earlier a comparison of found SNPs is not practical with the available data but it is seen from the copy numbers that 4219 genes in CHO DG44 are haploid versus 3586 in CHO DXB11. In addition, there are 44 deleted genes in CHO DXB11 versus 62 in CHO DG44. This difference can be ascribed to the harsh UV treatment that the cells were exposed to in the process of creating the CHO DG44 cell line compared to the relatively mild UV treatment of the CHO DXB11 cell line. The availability of additional sequence information from CHO-S and CHO DG44 among other would greatly improve the possibilities for comparing the genomic differences across a wider range of different CHO cell lines in the coming decades. This comparison could be highly informative about the evolutional path and diversity that exists across CHO cell hosts.

Analysis for enrichment of GO-terms revealed that a large portion of the changes in e.g. transcription factor copy number must have occurred in the early CHO cell. It appears that CHO DXB11 and F435 have further amplified ~20 transcription factors and CHO DG44 has reduced approximately the same number. Further studies of the transcriptome and proteome should be able to reveal the effects of these genomic changes and link differences in genotype and phenotype.

## Conclusions

In this work we have described the full genome sequencing of CHO DXB11 including SNPs and CNVs which differ between this cell line and the other CHO genomes that have been sequenced to date. The DHFR negative phenotype of the cell line was verified based on the lack of one allele and a missense mutation in the other transcriptionally active allele. The analysis of the CNVs revealed a large number of genes that were found to be haploid in the CHO cell lines which is important for correct SNP detection and detection strategy for knock out verification. It furthermore revealed unique patterns for the evolution of each of the chromosomes from the Chinese hamster to each of the sequenced CHO cell lines with chromosome one and four showing the lowest level of change.

## Methods

### Cell culture and genome extraction

CHO DXB11 cells were thawed from an in-house master cell bank. The cell bank was generated in 2000 from a vial of CHO DXB11 from L.A. Chasin, Columbia University. The cells were passaged in alpha MEM media with 10% FBS, 1% NEAA, 1% P/S. A second in-house suspension culture adapted CHO DXB11 cell line transfected with a plasmid encoding coagulation factor VIII coupled to *dhfr* was grown in HyClone CDM4CHO media supplemented with 1% Penicillin/Streptomycin and 100 nM MTX. Genomic DNA from both cell lines was extracted from 2 mio cells using DNeasy Blood & Tissue Kit (Qiagen) following manufacturer’s instructions. gDNA library and next-generation sequencing were performed by AROS Applied Biotechnology (Aarhus, Denmark) in a Illimina Hisq 2000 system for paired-end sequencing.

### NGS data treatment

The FASTQC tool (www.bioinformatics.bbsrc.ac.uk/projects/fastqc/) was used to evaluate the quality of the fastq files before and after treatment. The FASTX Toolkit (http://hannonlab.cshl.edu/fastx_toolkit/) was used to remove the adaptamers (fastx_trimmer) and trim the ends for bps with a quality score lower than 20 (fastq_quality_trimmer). An in-house algorithm was used to intersect the read-pairs after quality trimming. The reads were aligned to the *C. griseus* genome (downloaded from Genbank as assembly GCF_000419365.1) using BWA (version 0.6.2). The RealignerTargetCreator from GATK (version 1.6) was used to realign the reads in problematic regions and duplicate reads were removed using Picard MarkDuplicates (http://picard.sourceforge.net/).

The depth of reads at each position on the genome for identification of deleted genes was calculated using genomeCoverageBed from BEDTools (version 2.16.2). The depth for each gene was found by extracting the depth for each position in the coding region subsequently calculating the median using a custom script. The depths of each gene were normalized by 0.5x the median depth of all genes. The data were analyzed in R [29]. The depths are listed in Additional file 1: Table S5 and an overview of all genes in *C. griseus* with Genbank IDs, GC content and chromosome number is listed in Additional file 1: Table S6. The aligned reads from CHO DXB11 to the *C. griseus* genome was uploaded to the SRA under experiment ID: SRX689758. For direct download of raw reads from all public CHO cell lines currently in the SRA see Additional file 1: Table S1.

### SNP calls

SNPs were detected by samtools mpileup and bcftools. SNPs present in the coding region were found using CLC genomic workbench (CLC Bio, version 7). Haploid SNPs were detected by the filter: A minimum depth of 0.25x the median depth measured in the CDS regions, 90% of the reads calling the SNP had to differ from the reference sequence. Heterologous SNPs: A minimum depth of 0.75x the median depth measured in the CDS regions, a minimum of 40% of the reads should agree with the reference and 40% with the called allele. All SNPs interspaced by less than 5 bp to another SNP or an indel were filtered away as well as SNPs found when aligning the raw *C. griseus* reads to the *C. griseus* genome. Mutational bias was calculated by extracting reference and non-reference bases from the filtered SNP files and calculating the occurrence of different nucleotide transitions. Significance of bias were calculated as standard student t-test assuming equal variance comparing the observations for GC → AT/AT → GC for CHO-K1 PF, CHO-K1 SF, CHO-K1 ECACC and CHO-K1 ATCC versus CHO DXB11 and F435.

### Phylogenetic tree based on CNV data

Raw sequencing reads from CHO-K1 [3] and other CHO cell lines [12] were downloaded from the SRA (Additional file 1: Table S1). Reads were trimmed and intersected as described above and aligned to the *C. griseus* genome using BWA. Depths were estimated for each gene in each cell lined as described above and the occurrence of CNVs were estimated as the number of genes differing by more than 0.95 in normalized sequencing depth between two cell lines. A distance matrix were calculated, a phylogenetic tree was constructed using R with the package ape and phangorn using the neighbour joining algorithm. The tree was bootstrapped 100 times and the consensus tree was used.

### Gene copy number

The absolute copy number for each gene in each cell line was calculated as above by normalizing the read depth to the median. Genes were considered to be deleted if the read depth were 0 in a given cell line but > 0.95 in *C. griseus* (as 159 genes were ~0 in all cell lines incl. *C. griseus*). Haploid: depth higher than 0 but lower than 1.3 based on local minimum in CHO DXB11 between the haploid and diploid peak. Diploid: higher than 1.3 and lower than 2.7 based on local minimum in CHO DXB11 between the diploid and triploid. Triploid or higher: depth higher than 2.7.

### Chromosomal changes

All genes from the *C. griseus* genome (assembly GCF_000419365.1) as listed in the genome annotation file were downloaded from Genbank. The chromosome sorted *C. griseus* genome was downloaded from Genbank as Accession APMK00000000. The scaffold name and chromosome number was extracted from the fasta header. All genes were blasted against the chromosome sorted genome and the best hit (cutoff E-value = 0.05) was used as indicator for the chromosomal location.

### RNA sequencing

In order to deduce the transcriptional activity of *dhfr* from F435, RNA was extracted. A sample was taken 48 hours into the cultivation from 2x10e6 cells and RNA was extracted using TRIzol (Invitrogen) and the RNeasy Cleanup kit (Qiagen) following manufacturer’s instructions. RNA integrity was confirmed on an Agilent 2100 Bioanalyzer using total RNA nano chips (Agilent technologies, Santa Clara, Ca, USA). RNA concentration was measured using a NanoDrop spectrophotometer (NanoDrop Technologies). Multiplexed cDNA library generation and next-generation sequencing were performed by AROS Applied Biotechnology (Aarhus, Denmark) in an Illumina Hiseq 2000 system for paired-end sequencing. The FASTQC tool (http://www.bioinformatics.babraham.ac.uk/projects/fastqc/) was used to evaluate the quality of the fastq files before and after treatment. The FASTX Toolkit (http://www.bioinformatics.babraham.ac.uk/projects/fastqc/) was used to remove the adaptamers (fastx_trimmer) and trim the ends for base pairs with a quality score lower than 20 (fastq_quality_trimmer). An in-house algorithm was used to intersect the read-pairs after quality trimming. The reads were aligned to the CHO-K1 genome (downloaded from Genbank as assembly GCF_000223135.1) using tophat2 [30]. RNA expression from each of the genes on the NW_003614442.1 scaffold containing *dhfr* was calculated using genomeCoverageBed from BEDTools (version 2.16.2) and the mean expression level was used as indicator for expression.

### GO-term enrichment

A list of GO-terms associated with the CHO genome was downloaded from CHOgenome.org (http://www.chogenome.org/files/CHO_GO_Functions_12Sep13.txt), the list was rearranged and imported into R. Fisher’s exact test were used to find GO-terms enriched for either reduced or amplified genes for each of the CHO cell lines compared to *C. griseus* by > 0.95 difference in normalized copy number. All GO-terms, which had a p-value < 0.01 in just one cell line for either amplification or reduction in CN were included. Data listed in Additional file 1: Table S7.

## Additional files

Additional file 1: Table S1. SRA. **Table S2.** SNP qeneoverview. **Table S3.** Exon SNP’s DXB11. **Table S4.** Exon SNP’s K1ATCC. **Table S5.** Sequencing depth. **Table S6.** Cqriseus overvie. **Table S7.** GO-terms. Additional file 2: Figure S1. Read depth analysis of the 20661 in the C. griseus genome. For most of the genomes distinct peaks can be seen for genes present in one, two and three copies. Shoulders can be seen in the graphs for the cell lines sequenced at a lower depth. Only one peak is seen in wild type C. griseus as expected. **Figure S2.** The normalized sequencing depth of each gene in F435 and CHO DG44. Top left plot shows the distribution across all chromosomes. Compared to Figure 2 this reveals a much larger difference in CN. **Figure S3.** Distribution of CN across chromosomes for each cell line. **A)** Percentage of haploid genes **B)** percentage of diploid genes **C)** Percentage of genes, which are triploid or higher. **Figure S4.** Significant GO-terms in correlation to changes in CN Visualization of the 135 GO-terms, which are either significantly enriched in genes with CN reductions or amplifcations (Fisher's exact test). GO-terms are visualized in dark blue (p-value < 0.01), light blue (p-value < 0.05) or white (p-value > 0.05). Data attached in Additional file 1: Table S7.
