# Supplementary material for: Sequencing the CHO DXB11 genome reveals regional variations in genomic stability and haploidy
Source: BMC Genomics. 2015 Mar 8;16(1):160. doi: 10.1186/s12864-015-1391-x (PMC4359788; doi:10.1186/s12864-015-1391-x)
Supplement: Additional file 2: Figure S1. — Read depth analysis of the 20661 in the C. griseus genome. For most of the genomes distinct peaks can be seen for genes present in one, two and three copies. Shoulders can be seen in the graphs for the cell lines sequenced at a lower depth. Only one peak is seen in wild type C. griseus as expected. Figure S2. The normalized sequencing depth of each gene in F435 and CHO DG44. Top left plot shows the distribution across all chromosomes. Compared to Figure 2 this reveals a much larger difference in CN. Figure S3. Distribution of CN across chromosomes for each cell line. A) Percentage of haploid genes B) percentage of diploid genes C) Percentage of genes, which are triploid or higher. Figure S4. Significant GO-terms in correlation to changes in CN Visualization of the 135 GO-terms, which are either significantly enriched in genes with CN reductions or amplifcations (Fisher's exact test). GO-terms are visualized in dark blue (p-value < 0.01), light blue (p-value < 0.05) or white (p-value > 0.05). Data attached in Additional file 1: Table S7. [file 12864_2015_1391_MOESM2_ESM.docx]

## Supplementary materials


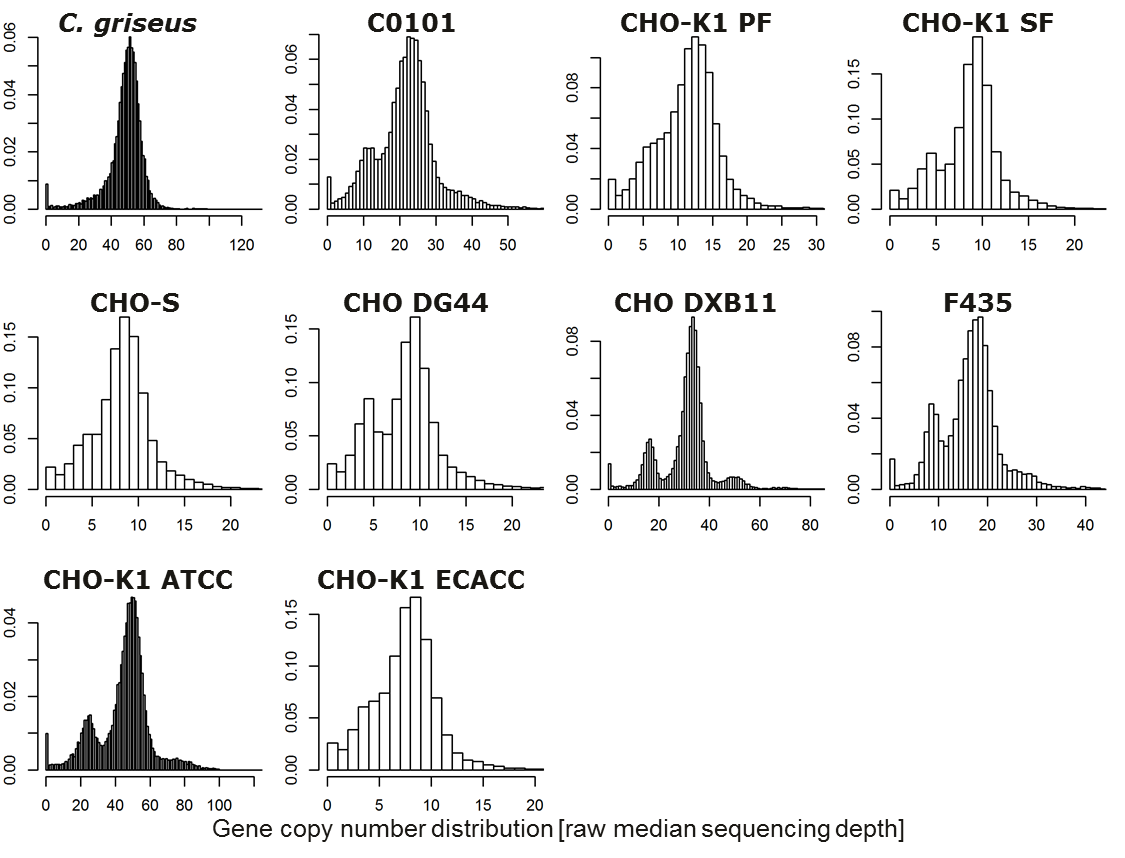
Figure S1 Read depth analysis of the 20661 in the *C. griseus* genome.

For most of the genomes distinct peaks can be seen for genes present in one, two and three copies. Shoulders can be seen in the graphs for the cell lines sequenced at a lower depth. Only one peak is seen in wild type *C. griseus* as expected.


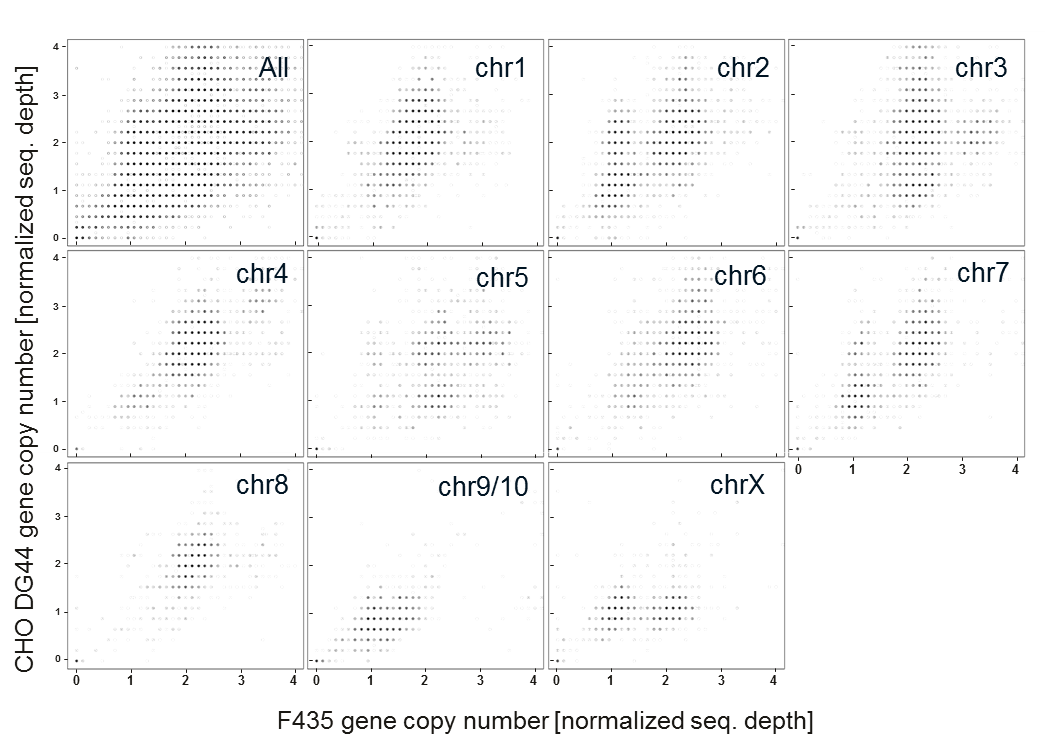


Figure S2 The normalized sequencing depth of each gene in F435 and CHO DG44.

Top left plot shows the distribution across all chromosomes. Compared to Figure 2 this reveals a much larger difference in CN.


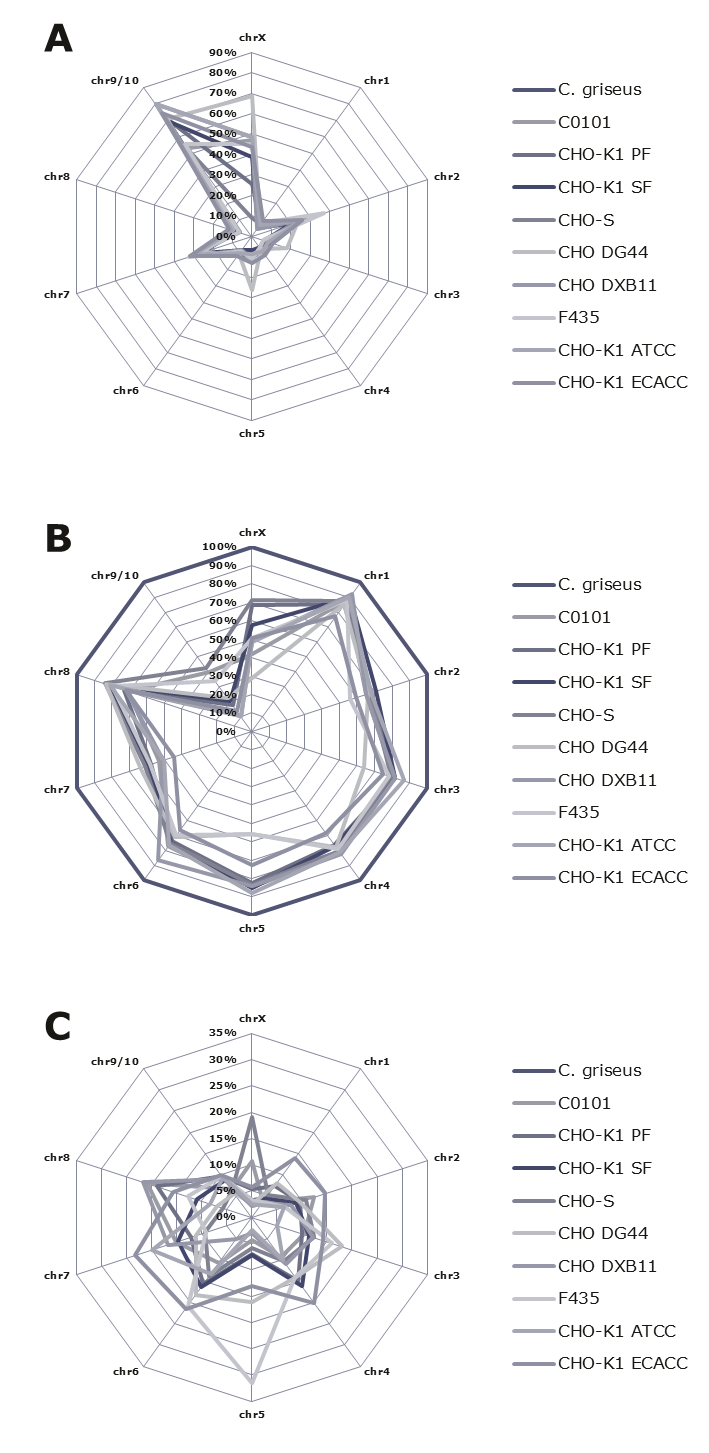


Figure S3 Distribution of CN across chromosomes for each cell line.

A) Percentage of haploid genes B) percentage of diploid genes C) Percentage of genes, which are triploid or higher.


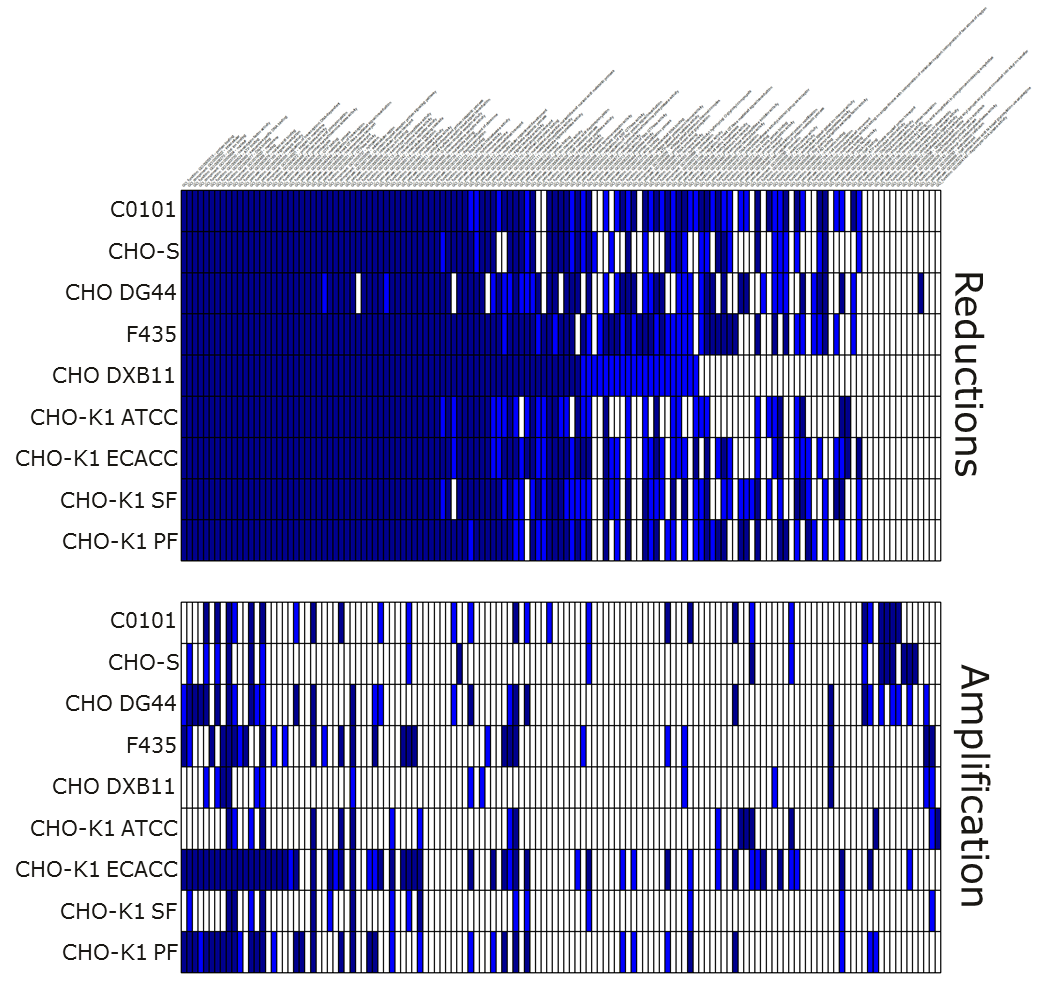


Figure S4 Significant GO-terms in correlation to changes in CN

Visualization of the 135 GO-terms, which are either significantly enriched in genes with CN reductions or amplifcations (Fisher's exact test). GO-terms are visualized in dark blue (p-value < 0.01), light blue (p-value < 0.05) or white (p-value > 0.05). Data attached in Supplementary table 7.
